# Brain Network Oscillations During Gait in Parkinson’s Disease

**DOI:** 10.3389/fnhum.2020.568703

**Published:** 2020-10-23

**Authors:** Doris D. Wang, Julia T. Choi

**Affiliations:** ^1^Department of Neurological Surgery, University of California, San Francisco, San Francisco, CA, United States; ^2^Department of Applied Physiology and Kinesiology, University of Florida, Gainesville, FL, United States

**Keywords:** locomotion, deep brain stimulation, basal ganglia, cerebral cortex, coherence, neurorehabilitation, closed-loop stimulation

## Abstract

Human bipedal walking is a complex motor task that requires supraspinal control for balance and flexible coordination of timing and scaling of many muscles in different environment. Gait impairments are a hallmark of Parkinson’s disease (PD), reflecting dysfunction of cortico-basal ganglia-brainstem circuits. Recent studies using implanted electrodes and surface electroencephalography have demonstrated gait-related brain oscillations in the basal ganglia and cerebral cortex. Here, we review the physiological and pathophysiological roles of (1) basal ganglia oscillations, (2) cortical oscillations, and (3) basal ganglia-cortical interactions during walking. These studies extend a novel framework for movement of disorders where specific patterns of abnormal oscillatory synchronization in the basal ganglia thalamocortical network are associated with specific signs and symptoms. Therefore, we propose that many gait dysfunctions in PD arise from derangements in brain network, and discuss potential therapies aimed at restoring gait impairments through modulation of brain network in PD.

## Introduction

Human gait requires the coordination of multiple brain areas to drive this complex and dynamic behavior. The supraspinal control of human locomotion begins in the cortical regions of supplemental motor area (SMA), premotor (PM) and primary motor (M1) cortices, where precise, intentional motor programs reach the basal ganglia for refinement, and then to the mesencephalic locomotor region (MLR), where cerebellar inputs join the MLR and descends to the medullary and pontine reticular formations to pass information to the spinal cord (Takakusaki, 2017). Disruptions in any part of this locomotion network can cause gait impairments. In the case of Parkinson’s Disease (PD), lack of dopaminergic innervation to the striatum not only affects the basal ganglia, but also causes brain network dysfunctions in many nodes of the circuit involved in locomotion.

The ability to directly measure neural activity on the population level gives investigators a unique opportunity to study neuronal network functions and dysfunctions in disease states. Synchronized activity patterns driven by neural ensembles can be measured in the form of membrane potential oscillations, and are becoming increasingly recognized as a means to drive dynamic brain coordination (Llinas, 1988; Buzsaki and Draguhn, 2004). Neural networks can oscillate across many frequencies ranges, and different oscillatory bands have been associated with distinctive behavioral states (Csicsvari et al., 2003; Hammond et al., 2007). Brain oscillations are important for nervous system functions because they provide key mechanisms for the encoding, storage, and processing of information across the neural network by biasing the probability of neuronal spiking activity (Llinas, 1988; Fries, 2005). These brain oscillations can be readily measured in the form of field potential by a wide variety of electrodes available for human clinical and research use, from local field potentials (LFP) recorded using depth electrodes penetrating the brain, electrocorticography (ECoG) potentials using electrode strip or grids placed over the surface of the brain, to electroencephalography (EEG) potentials using non-invasive scalp electrodes.

Using these methods over the past 15 years, researchers have developed a new framework for understanding neurological diseases resulting from disorders of network dynamics, or circuitopathies (Lozano and Lipsman, 2013). This work has generated a model for Parkinson’s disease where specific signs and symptoms are related to specific abnormal oscillatory synchronization in the basal ganglia-thalamocortical network. Extending this framework, we propose that dysfunctional oscillations in locomotor network contribute to different aspects of gait impairments in Parkinson’s disease. Here, we review recent evidence regarding the role of basal ganglia oscillations, cortical oscillations, and basal ganglia-cortical interactions during normal and abnormal gait in PD, and propose new therapeutic strategies to treat these circuitopathies to improve gait function in PD.

## Basal Ganglia Oscillations During Gait

Excessive basal ganglia oscillatory activity across different frequency bands have been associated with different motor signs of movement disorders. Increases in theta (4–8 Hz) and alpha (8–12 Hz) frequencies have been shown in the subthalamic nucleus (STN) of PD patients during resting tremor (Wang et al., 2005) and in the globus pallidus (GP) of patients with isolated dystonia at rest (Silberstein et al., 2003; Liu et al., 2008; Weinberger et al., 2012; Wang et al., 2018) and during phasic muscle contraction (Chen et al., 2006; Sharott et al., 2008). Elevated beta oscillations (13–30 Hz) and beta bursts (Tinkhauser et al., 2017b; Tinkhauser et al., 2018) has been observed in both the STN and GPi of PD patients at rest, which represents an akinesia state (Brown, 2003; Oswal et al., 2013; Wang et al., 2018). Additionally, narrowband gamma oscillations (60–80 Hz) has been found in the STN of PD patients with dopaminergic medication and during movement, and is associated with dyskinesia, a hyperkinetic state, in PD (Swann et al., 2016).

**FIGURE 1:**
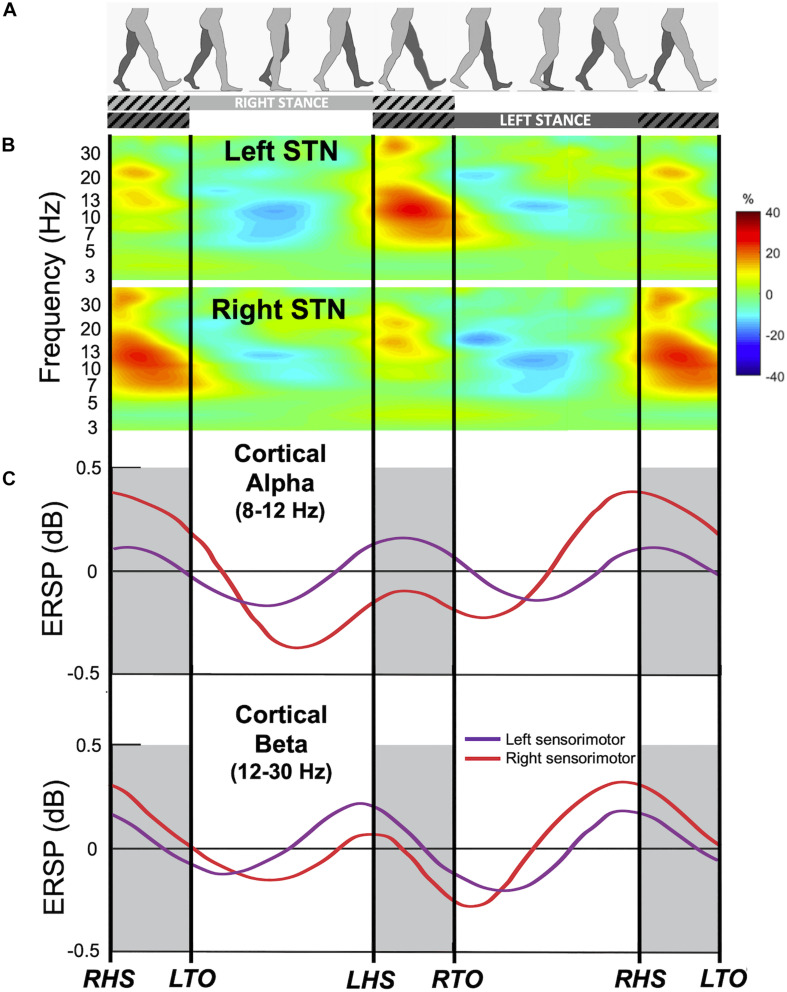
Gait event-related spectral changes from the subthalamic nucleus (STN) and sensorimotor cortex. **(A)** Schematic of gait cycle events for the right (light gray) and left leg (dark gray) during walking. Hatched lines indicate double support period when both legs are in contact with the ground. **(B)** Group time-frequency spectral analysis of field potentials recorded from the bilateral STN of 10 freely walking PD patients aligned to the gait cycle. Upper panel: contralateral averaged signals (i.e., Left STN time locked to right leg events and vice versa) demonstrating event-related modulations in alpha and beta frequency bands during gait. Lower panel: ipsilateral averaged signals (i.e., right STN time locked to right leg events). Color bar indicate percent change relative to average gait baseline. Modified from Hell et al. (2018) with permission. **(C)** Gait event-related spectral perturbation recorded from EEG of 8 healthy individuals during treadmill walking. Upper panel: average of the alpha (8–12 Hz) band amplitude from the left sensorimotor cortex (purple) and right sensorimotor cortex (red) time-locked to the gait cycle. Bottom panel: average of the beta (12–30 Hz) band amplitude. ERSP, event-related spectral perturbation; RHS, right heel strike; RTO, right toe off; LHS, left heel strike; LTO, left toe off. Modified from Gwin et al. (2011) with permission.

What happens to these oscillations during gait in PD? Normal upright walking consists alternating stance (foot is in contact with ground) and swing (foot is in the air) phase on each leg (Figure 1A); the left and right legs maintain reciprocal, out-phase-phase coordination that is critical for stable bipedal gait. Until recently, majority of LFP recordings are obtained from externalized DBS leads during the perioperative period via connection to an external amplifier, limiting patient mobility. Based on these limited stepping and walking tasks, several groups have reported alternating suppression of beta activity during stepping or gait in PD patients relative to the resting state. In a visually guided stepping task while sitting, alternating 20–30 Hz beta modulation was observed between left and right STN with gait cycle, and beta modulation increased with auditory cue (Fischer et al., 2018). Another study showed a suppression of beta power in the STN during both bicycling and walking, while this suppression was higher for bicycling (Storzer et al., 2017). Additionally, comparison between cued upper and lower extremity movements showed that there is greater movement-modulated desynchronization of high beta oscillations (24–31 Hz) for foot dorsi- and plantar-flexion compared to hand opening and closing (Tinkhauser et al., 2019). Now, with the availability of investigational neurostimulation devices with embedded sensing capabilities, new studies are shedding light on these basal ganglia oscillations change during gait in freely-moving PD patients. In a report of 10 patients implanted with such a bidirectional neurostimulator, it was found that high beta frequency power (20–30 Hz) and bilateral oscillatory connectivity are reduced during upright gait, as well as a reduction in overall high beta burst amplitude and burst lifetimes during gait compared to rest conditions (Hell et al., 2018; Figure 1B). However, not every study found decreased beta power during walking across all PD patients. Quinn et al. (2015) reported similar STN beta power during lying, sitting, standing, and forward walking for 14 akinetic-rigid (*n* = 7) and tremor-dominant (*n* = 7) PD patients, but only akinetic-rigid PD patients tended to exhibit beta desynchronization during walking, while tremor dominant patients did not. Another study found no difference in beta power, coherence, or cross-coupling during walking compared to sitting and standing; however, interhemispheric beta phase locking values decreased during walking compared to sitting and standing (Arnulfo et al., 2018). One explanation for this discrepancy on beta power among different studies is patient phenotypes, as akinetic-rigid PD patient could have greater gait-modulated beta changes (Quinn et al., 2015). Another explanation could be that since beta power appears to be modulated by phases of the gait cycle, averaging beta power for the duration of walking may not capture dynamic nature of this power modulation. Future studies characterizing the oscillatory changes during different phases of the gait cycle in different PD phenotypes will elucidate the dynamic control of gait within the basal ganglia.

Do these oscillatory changes represent physiological or pathological gait? In a study recording GPi LFP from patients with isolated dystonia without gait abnormalities, LFP power in the theta (4–8 Hz), alpha (8–12 Hz), and gamma (60–90 Hz) frequency bands was higher during walking on a treadmill than during either sitting or standing conditions, and beta (15–25 Hz) band was the only frequency that was down-regulated during walking (Singh et al., 2011). Subthalamic LFP also showed alpha, beta, and gamma frequency powers that are modulated and locked to the gait cycle (Hell et al., 2018). While in the latter study, the authors posit that these changes may be due to movement-induced artifacts, we believe these gait-cycle related oscillatory changes from the basal ganglia represent physiological modulations during gait as similar patterns of activity have been observed in PD patients during intraoperative stepping tasks, as well as in freely moving dystonia patients without gait symptoms, using both recordings from externalized leads and implanted sensing devices. Additionally, there are differences within the oscillatory characteristics in PD patient during effective walking vs. pathological walking such as freezing of gait (FoG). Direct measurements of STN LFPs of PD patients demonstrated differences in beta frequency amplitude (Toledo et al., 2014; Hell et al., 2018) and alpha frequency entropy (Syrkin-Nikolau et al., 2017) between patients with or without FoG, with high beta amplitude and alpha entropy in freezers. Another measurement of beta frequency activity, beta burst duration, were also found to be prolonged during FoG episodes and shortened by DBS which improved gait (Anidi et al., 2018). Together, these data suggest that during normal walking, the human basal ganglia produces precisely timed modulation of low band frequency and beta frequency bands which are disrupted during pathological gait patterns seen in PD.

## Cortical Oscillations During Gait

The human motor cortex exhibits rhythmic EEG activity during walking that is coupled to the gait cycle (Gwin et al., 2011; Petersen et al., 2012). Electrocortical sources in the anterior cingulate, posterior parietal, and sensorimotor cortex showed increased alpha- and beta-band power during the double support period (near the end of stance phase of each leg when both feet are on the ground), and this pattern of alpha- and beta-band power fluctuations alternate between the left and right hemispheres by half a gait cycle (Gwin et al., 2011; Figure 1C). In addition, lower gamma band activity (25–40 Hz) in the premotor cortex shows desynchronization around the time of heel contact during robot assisted walking (Wagner et al., 2012). Synchronization in the 40–200 Hz during normal walking compared with standing has also been reported in 2 subjects utilizing ECoG over leg M1 (McCrimmon et al., 2018).

What is the functional role of these cortical oscillations during gait? In comparison to basal ganglia oscillations during gait, cortical oscillations show a similar pattern of alternation between the left and right hemispheres, as well as alpha and beta suppression during normal walking relative to rest. Cortical oscillations also show spectral power modulation that are associated with specific phases of the gait cycle, according to the specific task demands. Gait-modulated alternating suppression of alpha and beta oscillations likely exerts the similar effect as those involved with upper extremity tasks in order to suppress unwanted motor information and drive motor cortical activity in a spatiotemporal specific manner (Miller et al., 2007; Miller et al., 2010; Yanagisawa et al., 2012; Stolk et al., 2019). Increased activity in the anterior cingulate during double support period (when the leading leg contacts the ground) is thought to reflect error processing (Gwin et al., 2011). In general, a reduction in alpha- and beta-band power, along with greater theta power, in the sensorimotor cortex during more demanding walking tasks (e.g., walking on a balance beam, speed matching) is thought to reflect greater cortical involvement (Sipp et al., 2013; Bulea et al., 2015; Nordin et al., 2020). Distinct beta oscillations during gait adaptation (step shortening and lengthening) is thought to serve different motor and cognitive processes: motor cortical alpha- and beta-band power decrease is thought to reflect anticipatory processes and movement execution, while frontal beta-band activity increase is thought to reflect cognitive top-down control (e.g., motor inhibition during step shortening) (Wagner et al., 2016).

Additionally, oscillatory activity from the motor cortex may directly facilitate ankle dorsiflexors in swing phase, and ankle plantorflexors in stance phase, during treadmill walking (Petersen et al., 2012; Jensen et al., 2018, 2019). Corticomsucular coherence between Cz and the tibialis anterior muscle (ankle dorsiflexor) in the 24–40 Hz frequencies has been detected during the swing phase of treadmill walking, and the amount of beta and gamma coherence increases when stepping on virtual targets displayed on screen, indicating greater cortical involvement during precision stepping (Jensen et al., 2018; Spedden et al., 2019). The timing of motor cortex activity in relation to the step cycle suggests that the motor cortex is important for the execution of gait modifications, and groups of pyramidal tract neurons may control synergistic muscles that are simultaneously active at different phases of the gait cycle (Drew and Marigold, 2015). In contrast, modulation of the supplementary motor area, premotor cortex and posterior parietal cortex EEG occurs well in advance of visually guided modifications during treadmill walking (Nordin et al., 2019), and during the anticipatory postural adjustments for gait initiation (Varghese et al., 2016), consistent with their role in the planning of visually guided locomotion (Drew and Marigold, 2015).

How do these cortical oscillations change during gait in PD? One study showed a large increase in theta activity over the vertex (Cz electrode) during freezing compared to normal walking, as well as an increase in beta band activity in the parietal lead (Shine et al., 2014). Another study showed increased synchronization in the theta, alpha, beta and gamma bands between the left and right hemispheres during walking in PD compared to age-matched controls, and that hyper-synchronization in the frontal lobe is associated with freezing of gait (Miron-Shahar et al., 2019). This suggests that increased oscillatory synchronization in the both cerebral cortex and basal ganglia are associated with clinical features of gait disorders in PD. Several studies have also examined corticomuscular coherence during walking and cycling in PD (Yoshida et al., 2018; Gunther et al., 2019; Roeder et al., 2020). Subjects with PD and older age-matched participants did not show significant differences in corticomuscular coherence during double support phase in walking, nor bilateral cycling (Yoshida et al., 2018; Roeder et al., 2020). Together, these studies suggest abnormal cortical oscillations in PD does not affect the direct corticospinal drive to muscles during walking, but may impair walking via other indirect pathways (e.g., cortical-basal ganglia loop, cortico-brainstem). In the next section, we explore evidence how cortical-basal ganglia network changes are associated with gait disorders in PD.

## Basal Ganglia-Cortical Interactions During Gait

Because many of the motor symptoms in PD arise from pathological synchronization across different frequency ranges throughout the basal ganglia-cortical network, it is important to understand how cortical-subcortical structures interact during gait in PD, and how these interactions may be disrupted during abnormal gait. In a study that investigated 7 PD patients with bilateral STN DBS, 5 of which had FoG, investigators were able to simultaneously record subthalamic LFPs and scalp EEG during walking through a course that induced FoG episodes (Pozzi et al., 2019). Interestingly, the authors found no difference in STN low frequency (theta, alpha) and beta power, beta burst duration, or interhemispheric STN coupling between effective walking and FoG. However, during normal walking, the cortex and STN were synchronized in a low frequency band (4–13 Hz). FoG was characterized by low frequency cortical-subthalamic decoupling at the transition from normal walking into gait freezing, was sustained during the freezing episode, and resolved with recovery of the effective walking pattern (Pozzi et al., 2019). This data suggests that during effective gait, cortical areas such as the SMA may be involved to produce normal gait patterns and transitions as a person is navigating through a path. Hypoactivation of cortical input may result in pathological oscillatory patterns in the basal ganglia that affect downstream locomotor regions. The specificity of cortical oscillatory changes during gait disorders in PD in relation to specific phases of the gait cycle has yet to be fully examined. Future studies focused on characterizing gait phase-specific cortical-basal ganglia oscillations in PD will provide a more complete understanding of the pathophysiology of PD gait.

## New Therapies to Modulate Brain Oscillations During Gait in PD

While conventional continuous DBS therapy has been used to improve many motor symptoms of PD, it has not significantly improved all aspects of gait and postural control. For instance, stimulation of the substantia nigra and pedunculopontine nucleus tend to improve anticipatory posture adjustments and gait postural control while having no significant effect on gait parameters, whereas STN or GPi DBS improve gait parameters and have heterogenous effects on postural control during gait initiation (Collomb-Clerc and Welter, 2015).

One possible reason that conventional DBS therapy fails to significantly improve postural adjustments and gait disturbances is that standard therapeutic stimulation occurs in a constant, open-loop manner, and does not account for the recursive and dynamic nature of gait, as shown by the cyclic oscillatory change that occur during the normal gait cycle. Closed−loop, or adaptive, DBS is a promising therapy for improving gait and balance functions in PD. Adaptive DBS automatically adjusts stimulation parameters based on brain signals that reflect different clinical states and pathophysiology. For instance, prototypes of adaptive DBS have used amplitude of STN beta oscillations (Little et al., 2013) and beta bursts (Tinkhauser et al., 2017a) to increase stimulation to reduce akinesia in PD. Others have used narrow-band gamma activity in the motor cortex, a biomarker for dyskinesia, to reduce side effects associated with STN stimulation (Swann et al., 2018). Therefore, understanding the neural signatures of gait in Parkinson’s disease – both in the cortex and in the basal ganglia – are critical for the development of better stimulation technologies.

**FIGURE 2:**
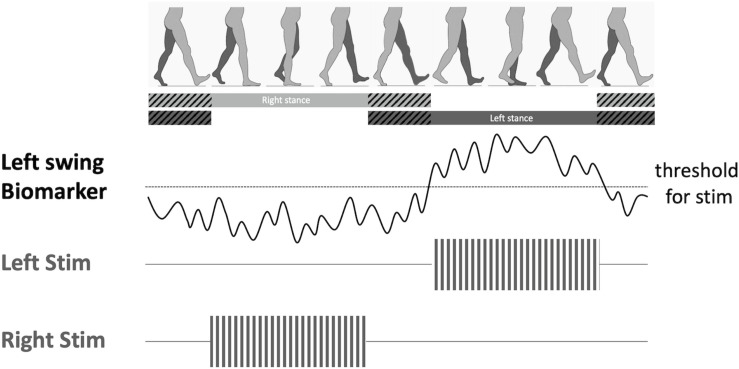
Proposed adaptive deep brain stimulation (aDBS) paradigm. When a gait phase-specific biomarker is discovered (i.e., swing phase), a bidirectional DBS device can decode the features of the biomarker in real time, and trigger stimulation once threshold is reached. This aDBS paradigm would allow alternating stimulation between the two brain hemispheres in order to synchronize stimulation to the swing phase of the right leg.

Based on this principal, alternating DBS stimulation between the two hemispheres of the brain has been proposed as a potential therapy to improve gait functions in PD (Fischer et al., 2018). As presented earlier, alternating patterns of synchronized field potentials have been recorded from the motor cortical regions (Seeber et al., 2015; McCrimmon et al., 2018) and the basal ganglia (Singh et al., 2011; Fischer et al., 2018; Hell et al., 2018) in healthy subjects, as well as in PD and dystonia patients without gait impairments, during normal walking. A potential new therapy would be to use these physiological biomarkers or external kinematic sensors of gait as control signals to stimulate the locomotor centers during specific phases of the gait cycle (Figure 2). By decoding the physiological brain signals associated with gait control, we can then develop adaptive DBS to recapitulate these normal brain network dynamics to restore gait functions in PD patients. For instance, we can use high beta (24–31 Hz) desynchronization, which is associated with lower-limb movement and gait (Hell et al., 2018; Tinkhauser et al., 2019), to trigger stimulation during contralateral leg swing during the gait cycle. Another possibility is to used pathological oscillations associated with gait disorders, such as prolonged beta bursts, to trigger stimulation during freezing of gait episodes (Anidi et al., 2018) to abort or prevent these pathological gait patterns. With technological advances in DBS hardware design, now it may be possible to implement such adaptive stimulation paradigms using bidirectional DBS interfaces that has both sensing and stimulation capabilities. These novel platforms will be crucial in understanding the dynamic neural control of gait, and designing temporally-specific patterns of neuromodulation to improve gait.

## Conclusion

Understanding brain oscillations during gait has theoretical significance because it will provide new mechanistic understanding of network dysfunctions during human locomotion in PD patients. Normal walking is associated with suppression of beta oscillations that exhibits a pattern of left-right alternation. Disorders of network dynamics in PD are manifested in elevated beta oscillations and excess interhemispheric phase locking during walking in PD patients with gait impairments. This new knowledge has tremendous translational potential as it will build a foundation for developing new neuromodulation therapies to improve gait in PD.

## Author Contributions

DW and JC contributed to the conceptual design, writing, editing, and generation of figures for this manuscript. All authors contributed to the article and approved the submitted version.

## Conflict of Interest

DW receives consulting fees from Medtronic and Boston Scientific Inc. The remaining author declares that the research was conducted in the absence of any commercial or financial relationships that could be construed as a potential conflict of interest.
