# Supplementary material for: Brain Network Oscillations During Gait in Parkinson’s Disease
Source: Front Hum Neurosci. 2020 Oct 23;14:568703. doi: 10.3389/fnhum.2020.568703 (PMC7645204; doi:10.3389/fnhum.2020.568703)
Supplement: Supplementary file 1 [file Table_1.DOCX]

**Supplementary Table**

| Study | Population | Recording site(s)/analysis | Task | Results |
| --- | --- | --- | --- | --- |
| Basal ganglia oscillations during gait in PD: | | | | |
| Fischer et al. (2018) | N=16 PD | Bilateral STN LFP | Stepping with or without auditory cue | Alternating 20-30 Hz beta modulation between left and right STN with gait cycle; beta modulation increased with auditory cu |
| Storzer et al. (2017) | N=13 PD (off med, DBS off-externalized leads) | STN LFP | Walking and bicycling | Greater beta power (13-35 Hz) suppression in bicycling relative to walking; patients with FOG exhibited a low beta power increase during movement |
| Tinkhauser et al. (2019) | N=12 PD (OFF med, intra-operative) | Bilateral STN LFP | Acoustically cued opening and closing of the hand; foot dorsiflexion and plantarflexion | Modulation in the lower beta band was similar between upper and lower limb tasks; but modulation of higher beta frequencies (24-31 Hz) was greater for leg movement |
| Hell, Plate, Mehrkens, and Botzel (2018) | N=10 PD | Bilateral STN LFP | Sitting, standing, walking | High beta frequency power (20-30 Hz) and bilateral oscillatory connectivity are reduced during gait; alpha, beta and gamma frequency power is modulated in time during gait, locked to the gait cycle. |
| Quinn et al. (2015) | N=15 PD classified as akinetic rigid or tremor dominant | Bilateral STN LFP | Sit, stand, walk | In akinetic rigid group, beta band power (13-30 Hz) during walking was lower during walking compared with resting stat |
| Arnulfo et al. (2018) | N = 8 PD (off med, off DBS) | Bilateral STN LFP | Sitting, standing, walking over ground | Interhemispheric beta (13-35 Hz) phase locking value decrease during walking compared to sitting and standing. No difference in beta power, coherence, or cross-coupling. |
| Singh, Levin, Mehrkens, and Botzel (2011) | N = 8 dystonia  (perioperative) | Bilateral GPi LFP | Walking on treadmill, standing, and sitting | LFP power in the theta (4–8 Hz), alpha (8–12 Hz) and gamma (60–90 Hz) frequency bands was higher during walking than during the resting conditions. Beta (15–25 Hz) frequencies were the only frequencies that were down-regulated during walking. |
| Syrkin-Nikolau et al. (2017) | N=14 PD (off med, off DBS) | Bilateral STN LFP | Stepping in place and walking with turning and barrier course | Freezers showed lower beta (13–30 Hz) power and greater beta Sample Entropy than Non-Freezers. During FOG, Freezers showed greater alpha (8–12 Hz) Sample Entropy than during walking without FOG. |
| Anidi et al. (2018) | N=12 PD (off med +/- DBS) | Bilateral STN LFP | Stepping in place and forward walking | Prolonged movement band beta burst durations were found during FOG and were shortened during DBS which improved gait. |
| Cortical oscillations during gait in PD: | | | | |
| Shine et al. (2014) | N=24 PD with FOG | EEG; cross spectrum analysis and cross frequency power ratio | Timed up-and go tasks | Episodes of freezing were associated with significant increase in theta band (4-8 Hz) power. Transition from. Normal to freezing of gait was associated with increase theta frequency coupling between Cz and Fz. |
| Miron-Shahar et al. (2019) | N=15 PD and N=8 control | EEG; interhemispheric phase synchronization | Standing, straight-line walking, and turning | PD show increased interhemispheric phase synchronization across alpha, beta and gamma bands during gait tasks. |
| Gunther et al. (2019) | N=9 PD (4 FOG+, 5 FOG-), N=4 older control | EEG and EMG; cross-correlation | Figure-eight shaped walking | Time-dependent cross-correlations between EEG and EEG amplitudes was increased at the beginning of stop and FOG episodes, especially in PD. |
| Roeder, Boonstra, and Kerr (2020) | N=24 PD, N=24 older healthy and N=24 younger healthy | EEG and EMG; corticomuscular coherence | Treadmill and over ground walking | Low beta (13-21 Hz) corticomuscular coherence is lower in PD compared to older healthy during double support phase. |
| Basal ganglia-cortical interactions during gait in PD: | | | | |
| Pozzi et al. (2019) | N=7 PD (off med, off DBS) | Bilateral STN LFP and cortical EEG | Walking along a path | Cortical STN synchronized in low frequency band (4–13 Hz) during effective walking. In contrast, gait freezing was characterized in every patient by low frequency cortical-subthalamic decoupling in the hemisphere with less striatal dopaminergic innervation. Of relevance, this decoupling was already evident at the transition from normal (effective) walking into gait freezing, was maintained during the freezing episode, and resolved with recovery of the effective walking pattern. |

**References:**

Anidi, C., O'Day, J. J., Anderson, R. W., Afzal, M. F., Syrkin-Nikolau, J., Velisar, A., & Bronte-Stewart, H. M. (2018). Neuromodulation targets pathological not physiological beta bursts during gait in Parkinson's disease. *Neurobiol Dis, 120*, 107-117. doi:10.1016/j.nbd.2018.09.004

Arnulfo, G., Pozzi, N. G., Palmisano, C., Leporini, A., Canessa, A., Brumberg, J., . . . Isaias, I. U. (2018). Phase matters: A role for the subthalamic network during gait. *PLoS One, 13*(6), e0198691. doi:10.1371/journal.pone.0198691

Fischer, P., Chen, C. C., Chang, Y. J., Yeh, C. H., Pogosyan, A., Herz, D. M., . . . Tan, H. (2018). Alternating Modulation of Subthalamic Nucleus Beta Oscillations during Stepping. *J Neurosci, 38*(22), 5111-5121. doi:10.1523/JNEUROSCI.3596-17.2018

Gunther, M., Bartsch, R. P., Miron-Shahar, Y., Hassin-Baer, S., Inzelberg, R., Kurths, J., . . . Kantelhardt, J. W. (2019). Coupling Between Leg Muscle Activation and EEG During Normal Walking, Intentional Stops, and Freezing of Gait in Parkinson's Disease. *Front Physiol, 10*, 870. doi:10.3389/fphys.2019.00870

Hell, F., Plate, A., Mehrkens, J. H., & Botzel, K. (2018). Subthalamic oscillatory activity and connectivity during gait in Parkinson's disease. *Neuroimage Clin, 19*, 396-405. doi:10.1016/j.nicl.2018.05.001

Miron-Shahar, Y., Kantelhardt, J. W., Grinberg, A., Hassin-Baer, S., Blatt, I., Inzelberg, R., & Plotnik, M. (2019). Excessive phase synchronization in cortical activation during locomotion in persons with Parkinson's disease. *Parkinsonism Relat Disord, 65*, 210-216. doi:10.1016/j.parkreldis.2019.05.030

Pozzi, N. G., Canessa, A., Palmisano, C., Brumberg, J., Steigerwald, F., Reich, M. M., . . . Isaias, I. U. (2019). Freezing of gait in Parkinson's disease reflects a sudden derangement of locomotor network dynamics. *Brain, 142*(7), 2037-2050. doi:10.1093/brain/awz141

Quinn, E. J., Blumenfeld, Z., Velisar, A., Koop, M. M., Shreve, L. A., Trager, M. H., . . . Bronte-Stewart, H. (2015). Beta oscillations in freely moving Parkinson's subjects are attenuated during deep brain stimulation. *Mov Disord, 30*(13), 1750-1758. doi:10.1002/mds.26376

Roeder, L., Boonstra, T. W., & Kerr, G. K. (2020). Corticomuscular control of walking in older people and people with Parkinson's disease. *Sci Rep, 10*(1), 2980. doi:10.1038/s41598-020-59810-w

Shine, J. M., Handojoseno, A. M., Nguyen, T. N., Tran, Y., Naismith, S. L., Nguyen, H., & Lewis, S. J. (2014). Abnormal patterns of theta frequency oscillations during the temporal evolution of freezing of gait in Parkinson's disease. *Clin Neurophysiol, 125*(3), 569-576. doi:10.1016/j.clinph.2013.09.006

Singh, A., Levin, J., Mehrkens, J. H., & Botzel, K. (2011). Alpha frequency modulation in the human basal ganglia is dependent on motor task. *Eur J Neurosci, 33*(5), 960-967. doi:10.1111/j.1460-9568.2010.07577.x

Storzer, L., Butz, M., Hirschmann, J., Abbasi, O., Gratkowski, M., Saupe, D., . . . Schnitzler, A. (2017). Bicycling suppresses abnormal beta synchrony in the Parkinsonian basal ganglia. *Ann Neurol, 82*(4), 592-601. doi:10.1002/ana.25047

Syrkin-Nikolau, J., Koop, M. M., Prieto, T., Anidi, C., Afzal, M. F., Velisar, A., . . . Bronte-Stewart, H. (2017). Subthalamic neural entropy is a feature of freezing of gait in freely moving people with Parkinson's disease. *Neurobiol Dis, 108*, 288-297. doi:10.1016/j.nbd.2017.09.002

Tinkhauser, G., Shah, S. A., Fischer, P., Peterman, K., Debove, I., Nygyuen, K., . . . Brown, P. (2019). Electrophysiological differences between upper and lower limb movements in the human subthalamic nucleus. *Clin Neurophysiol, 130*(5), 727-738. doi:10.1016/j.clinph.2019.02.011
